# Role of Excessive Autophagy Induced by Mechanical Overload in Vein Graft Neointima Formation: Prediction and Prevention

**DOI:** 10.1038/srep22147

**Published:** 2016-02-26

**Authors:** Ya-Ju Chang, Hui-Chun Huang, Yuan-Yu Hsueh, Shao-Wei Wang, Fong-Chin Su, Chih-Han Chang, Ming-Jer Tang, Yi-Shuan Li, Shyh-Hau Wang, Kirk K. Shung, Shu Chien, Chia-Ching Wu

**Affiliations:** 1 Institute of Basic Medical Sciences, National Cheng Kung University, Tainan, Taiwan; 2 Division of Plastic Surgery, National Cheng Kung University Hospital, Tainan, Taiwan; 3 Department of Biomedical Engineering, National Cheng Kung University, Tainan, Taiwan; 4 Medical Device Innovation Center, National Cheng Kung University, Tainan, Taiwan; 5 Department of Physiology, National Cheng Kung University, Tainan, Taiwan; 6 Institute of Engineering in Medicine, University of California, San Diego, CA92093, USA; 7 Department of Computational Science, National Cheng Kung University, Tainan, Taiwan; 8 Department of Biomedical Engineering, University of Southern California, Los Angeles, CA90089, USA; 9 International Research Center for Wound Regeneration and Repair, National Cheng Kung University, Tainan, Taiwan

## Abstract

Little is known regarding the interplays between the mechanical and molecular bases for vein graft restenosis. We elucidated the stenosis initiation using a high-frequency ultrasonic (HFU) echogenicity platform and estimated the endothelium yield stress from von-Mises stress computation to predict the damage locations in living rats over time. The venous-arterial transition induced the molecular cascades for autophagy and apoptosis in venous endothelial cells (ECs) to cause neointimal hyperplasia, which correlated with the high echogenicity in HFU images and the large mechanical stress that exceeded the yield strength. The *ex vivo* perfusion of arterial laminar shear stress to isolated veins further confirmed the correlation. EC damage can be rescued by inhibiting autophagy formation using 3-methyladenine (3-MA). Pretreatment of veins with 3-MA prior to grafting reduced the pathological increases of echogenicity and neointima formation in rats. Therefore, this platform provides non-invasive temporal spatial measurement and prediction of restenosis after venous-arterial transition as well as monitoring the progression of the treatments.

Vein graft bypass surgery is a frequently used clinical procedure for vascular reconstruction in treating cardiovascular diseases. However, 10–15% of restenosis occurs within one year after surgery, and almost half of the venous grafts fail after 10 years^1^,^2^,^3^. The magnitudes of shear stress is different in arteries (10–70 dyn/cm^2^) and veins (1–6 dyn/cm^2^)^4^. Mechanical mismatch of the vein and the artery results in pressure-induced bulging of the bypassed vein and subsequent graft failure^5^,^6^. Exposure of a venous bypass graft to the arterial circulation has been shown to be a stimulus for endothelium damage^7^,^8^. In normal vessels, endothelial cells (ECs) form a monolayer on the vessel wall to provide an interface between the blood stream and vascular tissues. Experimental grafting of LacZ-expressing veins to wild-type arteries showed the loss of β-galactosidase-positive ECs after the surgery^9^, which indicates the damage to venous endothelium by the arterial environments.

Autophagy is an evolutionarily conserved process to degrade damaged organelles within the double-membrane vacuoles inside lysosomes^10^. General stresses, such as starvation and bacterial infection, induce autophagy flux which protects ECs against injury by removing deleterious materials^11^. A window of optimal autophagic activity seems to be critical to the maintenance of cardiovascular homeostasis and function^12^. Autophagic flux involves increases in both the formation of the autophagosome and its fusion with the lysosome^13^. It has been shown that the maladaptive response in autophagic processes is correlated with cancer, aging, neurodegenerative disorders, and cardiovascular disease^12^,^14^,^15^. In atherosclerotic plaques, excessive autophagy results in the secretion of inflammatory cytokines and causes programmed cell death^14^. Excessive autophagy has been observed in the heart following cardiac pressure overload after transverse aortic constriction, which resulted in cardiac atrophy^16^. Little is known about the interrelations among autophagy, endothelial damage, and inflammatory responses in the pathological progression of vein graft disease.

Clinical cases to monitor the changes of blood flow and lumen size during stenosis were reported by computed tomography (CT), three-dimensional (3D) angiography, magnetic resonance image (MRI), and ultrasound images^17^,^18^,^19^. However, the continuous monitoring of blood flow and vascular structures during stenosis is difficult due to the limited image resolution. High-frequency ultrasound (HFU) in the range of 20–50 MHz provides enhanced image resolutions at the sub-tissue level (approximately 10^−5 ^m), which allows for visualizing detailed anatomical structures, and can be used for diagnostic, therapeutic, and surgical applications, especially in relation to cardiovascular interventions^20^. In addition, tissue inflammatory responses after fracture injury in mice are highly correlated with the alteration of echogenicity in HFU images^21^. Due to the increase in demand for following the prognosis of stenosis, the American Society of Echocardiography (ASE) and Society of Vascular Medicine and Biology have published the guidelines for using hemodynamic parameters acquired from color doppler to predict vein graft failure^22^. HFU may provide the high-resolution clinical hemodynamic measurements to predict graft restenosis. The von-Mises stress, an indication of wall tensile stress in vessels, showed more superior outcome predictions than simply measuring vessel diameter or flow^23^,^24^. Understanding the dynamics of cellular mechanical responses for autophagy formation induced by venous-to-arterial flow alteration in venous ECs is critical for the management of vein graft diseases. The current study uses live animal HFU images to compute the von-Mises stress and predict the occurrence of endothelial damage in the complex vascular structure. The histological evidence and molecular mechanism in inflammation and autophagy in vein graft failure are directly proven by *in vivo* and *ex vivo* experiments. Inhibition of excessive autophagy formation prevents the pathological initiation of vein graft failure. These results allow for the prediction of the mechanical responses in grafted veins and provide guidance for rational treatments.

## Results

### Endothelium damage and autophagy in vein graft disease

**Figure 1:**
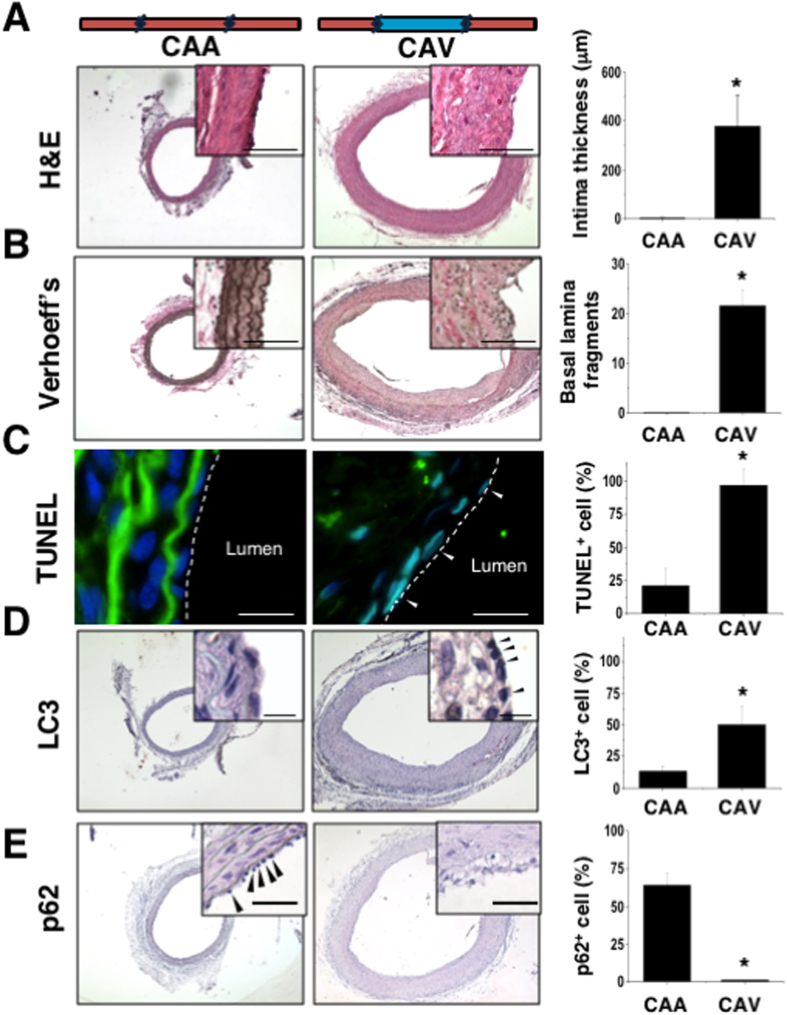
The animal model of vein graft stenosis was established by bridging the vein graft to carotid artery (CAV). The hematoxylin and eosin (H&E) staining showed a significant increase in neointima hyperplasia with the thickening of the intima layer in CAV rats (n = 12) (**A**). The arterial control surgery (CAA) did not have histological changes. Loss of the elastic fiber and fragments of the basal laminar were observed in the CAV graft using Verhoeff’s staining (n = 5) (**B**). Cellular apoptosis was detected using terminal deoxynucleotidyl transferase dUTP nick end labeling (TUNEL). An increase in cell death was then observed by counting TUNEL^+^ cells within the intimal layer (n = 8) (**C**). The excessive autophagy was demonstrated by induction of specific autophagy marker (LC3) (**D**) and degradation of p62/SQSTM1 expression (**E**) by immunohistological staining in CAV grafts (n = 8) (**D**). Scale bars in (**A**) and (**B**): 50 μm. Scale bars in (**C,D**): 20 μm. *Significant difference from the artery graft rats, p < 0.05.

To create an arterial-venous transition in carotid vessels (CAV) for investigating vein graft disease, the right external jugular vein (5 mm in length) was resected from Sprague-Dawley rats, and the two free ends were connected to the left common carotid artery of the same rats by end-to-end anastomosis with 10-0 sutures (Supplementary Fig. S1A). The carotid arterial-arterial (CAA) control anastomosis was created by resecting the left common carotid artery (5 mm in length) and then suturing the free ends back to the same artery. The vein graft was immediately reperfused with arterial blood flow by releasing the vessel clamp after surgery (Supplementary Fig. S1B). Three weeks after surgery, tissue samples from CAV and CAA grafts were collected. The CAV graft failure was demonstrated by tunica intima thickening in hematoxylin and eosin (H&E) staining (Fig. 1A) and elastic fiber degradation in Verhoeff’s staining (Fig. 1B). Both H&E and Verhoeff’s stainings showed significant stenosis, which was similar to the pathogenesis in human vein graft disease. Apoptosis analyses indicated the induction of EC death in CAV (Fig. 1C, white arrowhead), but not in CAA, as demonstrated by positively stained cells following terminal deoxynucleotidyl transferase dUTP nick end labeling (TUNEL) in the neointima near the lumen (dash line). Excessive autophagy formation was significantly induced in ECs of CAV in comparison to CAA, as indicated by the positive staining of intracellular microtubule-associated protein 1A/1B-light chain 3 (LC3) in neointima (Fig. 1D) and the decrease in p62 (also called SQSTM1, which is a marker for autophagic flux^25^) (Fig. 1E). These findings indicate the excessive autophagy in the CAV animals after 3 weeks of surgery. The occurrence of higher levels of cellular apoptosis and excessive autophagy in CAV suggests a disruption of cell homeostasis during the progression of stenosis.

### Prediction and validation of endothelium damage in vein graft

**Figure 2:**
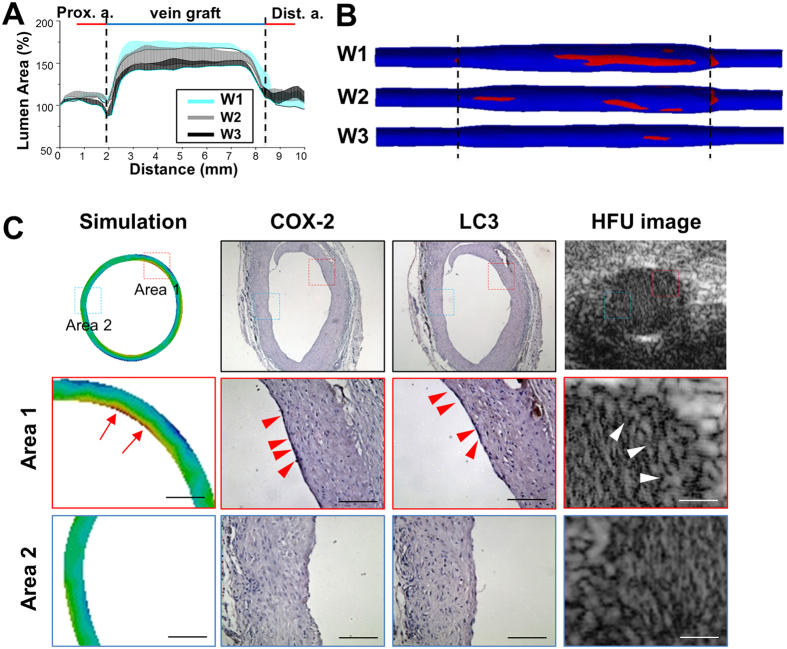
High spatial agreement of excessive autophagy and inflammation in the area with mechanical overload. Quantification of lumen area from high-frequency ultrasound (HFU) demonstrated the stenosis progression in living animals after surgery for 1, 2, and 3 weeks (W1, W2, W3, n = 8) (**A**). The potential damages of endothelial cells (EC) are predicted (red color) when the von-Mises stress exceeds the yield threshold (1000 Pa) (**B**). Immediately after the HFU scanning, the vessels at 3 weeks after surgery were harvested for histological analysis (**E**). We observed an excellent agreement with the predicted high-stress locations in simulation, increased expression of autophagy formation, increased COX-2 expression (arrowheads), and high echogenicity in HFU images (arrowheads). The marked squares indicated the areas of the enlarged images for high stress region (red square) and low stress region (blue square). Scale bar: 50 μm. *Significant difference from the results in the artery, p < 0.05.

To monitor the dynamic changes in vein graft restenosis at early stages in living animals, a 30-MHz HFU system was used to scan the detailed vascular structures (Supplementary Fig. S2A). The dynamic geometrical changes in the vein graft of the same rat after surgery at one (W1, Supplementary Video S1), two (W2, Supplementary Video S2), and three weeks (W3, Supplementary Video S3) were acquired by serial scanning of cross-section HFU images at 50-μm intervals, starting from the artery 2 mm proximal to the proximal suture site, through the entire graft vessel, and then to the artery 2 mm distal to the distal suture site. Increases in echogenicity in ultrasonography were observed on the vessel wall of CAV, thereby suggesting structural changes in the intima. The vessel lumen area, which was determined from the vessel border identified in serial HFU images, became progressively narrowed over the 3-week period after vein graft surgery (Fig. 2A).

The 3D vessel geometry was reconstructed and created a finite element vascular structure to simulate the biomechanical responses during vein graft stenosis. Because tissue/material can be damaged when mechanical stress (von-Mises stress) exceeds the yield stress, a yield stress of 1000 Pa^26^ was used to estimate the damage of endothelial cells and showed the potential damage sites in red color on the vascular wall to demonstrate the dynamic post-surgery changes from W1 to W3) (Fig. 2B). The elements in the vein grafts were exposed to higher stresses that exceeded the yield stress, especially during the first week after surgery.

To compare the localizations of predicted EC damage sites with those that were observed in the histological examination and the echogenicity in the HFU images, the HFU-scanned vein graft was immediately processed for computational simulation and immunohistological staining (Fig. 2C). Excellent agreement was observed between the results of simulation and ultrasonography, as well as histological findings. The EC damage sites predicted by yield stresses (arrowhead in simulation, >1000 Pa) agree well with those diagnosed by hyperechogenicity in HFU images (Fig. 2C, red square enlarged image of Area 1). Those locations (Area 1) also showed positive staining of cyclooxygenase-2 (COX-2) for the triggering of inflammation (arrowheads in COX-2) and excessive autophagy (arrowheads in LC3). The negative controls for the lower stresses were associated with lower echogenicity and negative stainings of COX-2 and LC3 in the same set of images (Fig. 2C, blue square enlarge image of Area 2). Therefore, the combination of HFU images and computational simulation provided a non-invasive platform to estimate the biomechanical remodeling in living animals. These areas with the overloading of stress on the venous endothelium can predict the endothelial damage sites that would later initiate pathological processes.

### Arterial flow induction of venous endothelium damage in *ex vivo* vessels

**Figure 3:**
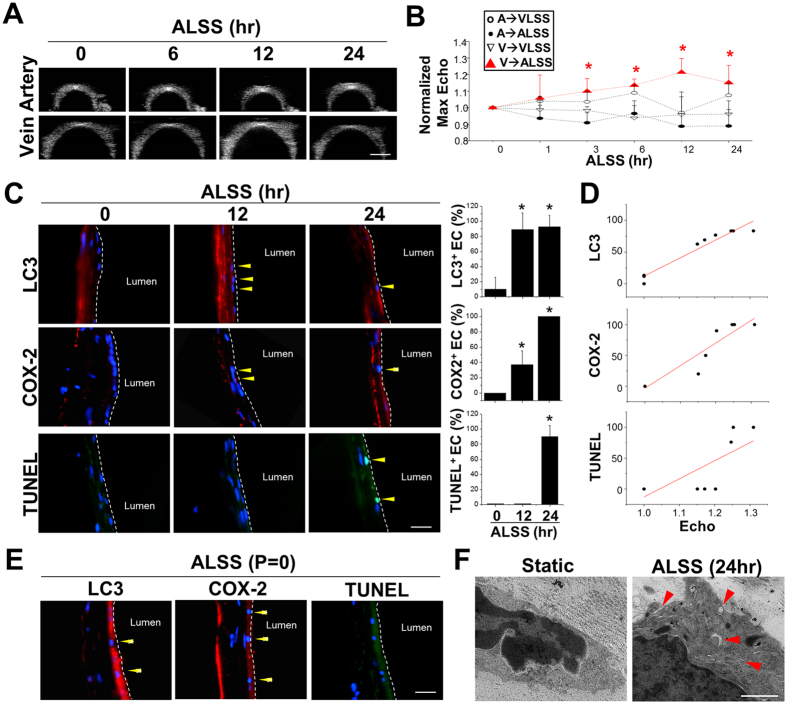
Detection of ALSS-induced vein damage in the *ex vivo* system. The HFU images of the isolated artery and vein perfused with arterial laminar shear stress (ALSS, 12 dyn/cm^2^) at 0, 1, 3, 6, 12, and 24 hrs (**A**). Quantification of the normalized maximum echogenicity (normalize to the same vessel at 0 hr) showed that ALSS increased the echogenicity when perfusing the vein for 3 to 24 hrs (max at 12 hrs), but not in the artery or when subjecting either artery or vein to VLSS (n = 5) (**B**). The immunofluorescence staining of cross-sectioned venous tissues demonstrated that ALSS induces venous EC autophagy formation at 12 and 24 hrs (red color for LC3 expression), COX-2 expression (mild level of red color at 12 hrs, and high at 24 hrs), and EC apoptosis at 24 hrs after ALSS application (green color for TUNEL positive cells) (n = 5) (**C**). Strong positive correlations of echogenicity were observed with LC3 and COX-2 expressions (n = 5) (**D**). The ALSS-induced autophagy in venous ECs was confirmed morphologically by the accumulation and formation of double-membrane autophagosomes (arrowheads) using transmission electron microscopy (**E**). Without arterial pressure, the ALSS still induced venous EC damage with accompanied by the positive staining of LC3, COX-2, and TUNEL (**F**). Scale bar: 20 μm. *Significant difference from the results at 0 h, p < 0.05.

To illustrate that shear stress transition has a major role in triggering venous endothelial damage and to confirm the HFU hyperechogenicity with autophagy-inflammation findings, the artery and vein were isolated from rats and subjected to arterial or venous shear stresses in the *ex vivo* perfusion system (Supplementary Fig. S3A). The HFU system was utilized to determine the echogenicity in veins under the *ex vivo* application of arterial laminar shear stress (ALSS, 12 dyn/cm^2^) or venous laminar shear stress (VLSS, 1 dyn/cm^2^) for 1, 3, 6, 12, and 24 hrs (Fig. 3A). The echogenicity normalized to that in the same vessel before shear stress stimuli was significantly increased in the vein after ALSS application for 3 hrs and was observed continuously between 3 to 24 hrs but not in the vein after VLSS, nor in the arteries subjected to either ALSS or VLSS (Fig. 3B). Concurrently, significant increases in autophagy formation (LC3 puncta, Fig. 3C) and continuous increases in COX-2 mediated inflammation (COX-2, Fig. 3C) were observed in venous ECs after being subjected to ALSS for 12 and 24 hrs, with venous EC apoptosis observed at 24 hrs (TUNEL, Fig. 3C). Our results indicate that ALSS triggers damage cascades consisting of COX-2 mediated inflammatory responses, autophagy, and cell death in the venous endothelium. The normalized max echogenicity (Fig. 3B) showed a positive correlation with LC3 and COX-2 stainings, thereby indicating that the HFU system can be used to detect autophagy-induced apoptosis and vessel inflammation (Fig. 3D). The detailed relationship between echogenicity in HFU images and the predictor variables in molecular responses were analyzed by multiple regressions (Table 1). The LC3-positive staining is the single predictor variable that shows the highest association (adj. R^2^) with echogenicity (dash underline). When considering multiple variables, the echogenicity may indicate the combination of excessive autophagy and cell death (highest adj. R^2^ as combine LC3 and TUNEL staining, underlined in Table 1).

The accumulation of typical autophagosomes with double membranes (arrowheads, Fig. 3E) was observed in venous ECs after 24 hrs ALSS perfusion using transmission electron microscope (TEM). To assess whether the induction of venous EC damage by venous-arterial flow transition was the result of increased shear stress or pressure, experiments were performed under the same shear stress but with different pressure levels acting on the ECs, by concomitantly adjusting the upstream and downstream pressure levels. Similar inductions of LC3, COX-2, and TUNEL expressions were observed in venous ECs that underwent ALSS perfusion in the absence of pressure (P = 0, Fig. 3F). When considered together, the increase in echogenicity in HFU images after subjecting the venous ECs to ALSS shows a strong positive correlation with EC damage in response to the induction of autophagy overactivation (Table 1).

### Alleviation of EC damage and neointimal hyperplasia by excessive autophagy inhibition

**Figure 4:**
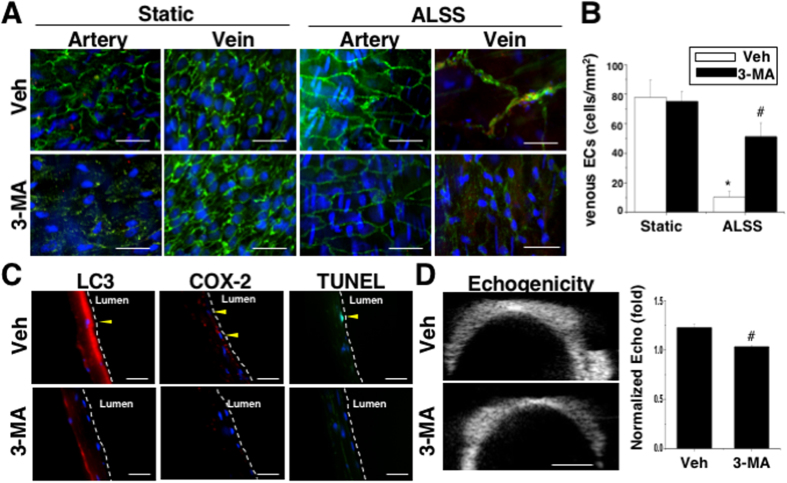
Transient inhibition of autophagy by 3-MA prevented the loss of venous ECs under ALSS. The detection of endothelium integrity and autophagy by *en face* staining of CD31 (green) and LC3 (red). The intact endothelial monolayer was observed in both arteries and veins under static conditions. After 24 hrs of ALSS stimulation, venous ECs were peeled off with high expressions of autophagy formation in venous ECs (n = 7) (**A**). Incubation of the vein with an autophagy inhibitor (3-MA) for 30 min before the application of ALSS significantly preserved venous ECs (n = 5) (**B**). 3-MA decreased ALSS-induced LC3-, COX-2-, and TUNEL-positive staining (n = 6) (**C**), and echogenicity (n = 4) (**D**) at 24 hrs. Scale bar in (**A**) 50 μm. Scale bar in (**C**) 30 μm. *Significant difference from the static condition, p < 0.05. ^#^Significant difference from the condition without 3-MA treatment, p < 0.05.

Results from both *in vivo* (Fig. 1) and *ex vivo* (Fig. 3) experiments suggest that excessive autophagy plays an important role in venous EC damage during the venous-arterial flow transition. To determine whether the ALSS-induced autophagy overactivation has an essential role in damaging the endothelium in vein grafts, the autophagic puncta and endothelial integrity were assessed by *en face* staining. Under static conditions, both arteries and veins showed intact confluent EC monolayers (shown by CD31 staining) with rare autophagosome formation (assessed by LC3 staining) (Fig. 4A). The arteries subjected to ALSS showed no induction of autophagy formation or alteration in endothelium integrity. However, the veins subjected to ALSS for 24 hrs showed excessive positive staining of LC3 clustered on the luminal surface of venous ECs, which underwent severe peeling off. Transient pretreatment (30 min before ALSS stimuli) with a specific autophagy inhibitor (3-methyladenine, 3-MA) attenuated LC3 clustering in venous ECs (Fig. 4A) and significantly rescued the endothelial detachment (Fig. 4B). The immunofluorescent staining of LC3 and COX-2 in frozen-sectioned samples further demonstrated that the 3-MA-pretreatment blocked autophagosome formation and reduced apoptosis in venous ECs subjected to ALSS (Fig. 4C), as well as inhibiting the ALSS-triggered hyperechogenicity in HFU images (Fig. 4D). Inhibition of ALSS-induced excessive autophagy using 3-MA-pretreatment was further confirmed by the p62/SQSTM1 expression in venous ECs (Supplementary Fig. S4). These results indicate that the transient inhibition of autophagy can block the inflammatory responses and apoptosis of venous ECs in response to ALSS.

**Figure 5:**
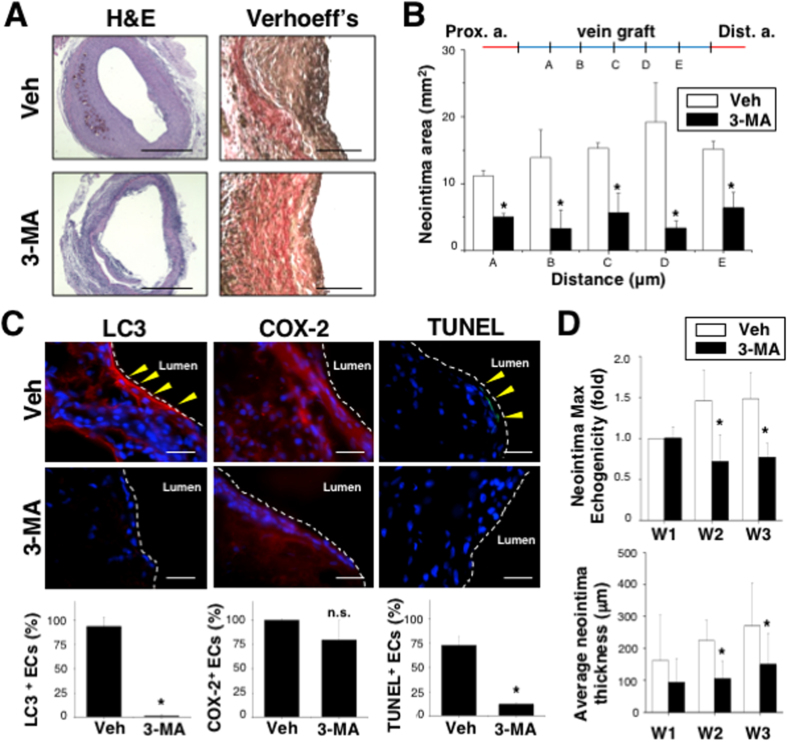
Pre-incubation of the venous segment with 3-MA for 30 min before grafting to the artery prevented neointima formation in rats. At 3 weeks after surgery, H&E and Verhoeff’s staining demonstrated a reduction of neointimal hyperplasia by 3-MA pretreatment in grafted veins (**A**). The significant decreases of the neointima area were quantified at different distances of the grafted veins (n = 6) (**B**). The 3-MA-pretreated vein grafts show inhibition of LC3- and TUNEL-positive cells (n = 6) (**C**). Decreases of hyperechogenicity and neointimal thickness were determined by HFU at W2 and W3 in the living rats that received 3-MA pretreatment (n = 6) (**D**). *Significant difference from the condition without 3-MA treatment, p < 0.05.

To investigate whether transient pretreatment with 3-MA could provide a long-term therapeutic effect to prevent neointimal formation in the vein graft after surgery, the vessel grafts were pre-incubated with 3-MA for 30 min (same pretreatment as in *ex vivo* experiments) before being sutured to the artery. The 3-MA pretreatment did inhibit the intimal hyperplasia of CAV (3 weeks after surgery), as demonstrated by H&E and Verhoeff’s staining (Fig. 5A). Quantification of the intimal hyperplasia area at different distances of the grafted veins from proximal (site A: ~1 mm from the proximal suture) to distal (site E: ~4 mm from the proximal suture) sections demonstrates that pre-incubation with 3-MA decreased neointimal thickness in the entire graft (Fig. 5B). Furthermore, pretreatment with 3-MA inhibited cellular apoptosis (TUNEL) and excessive autophagy (LC3), but had little effect on COX-2 expression, in the neointima area of vein grafts (Fig. 5C). p62/SQSTM1 was observed in the endothelium of vein graft with 3-MA pretreatment (Supplementary Fig. S5). The association of Beclin 1 to B-cell lymphoma (Bcl)-2 and Bcl-X(L) family members has been shown to inhibit autophagy and result in pro-survival signals^27^,^28^. Our results demonstrated that the dissociation of Beclin 1 complex from Bcl2 and Bcl-X(L) (Supplementary Fig. S6) in the grafted vein, while transient pretreatment with 3-MA prevented such dissociation, thereby suggesting that the Beclin 1 complex may play a significant role for EC survival. The scanning of HFU images was performed to monitor the dynamic changes in vascular structures in 3-MA-treated grafts after CAV surgery for 1, 2, and 3 weeks (Supplementary Fig. S7). The quantified results show that 3-MA pretreatment decreased the neointimal thickness and echogenicity, particularly at W2 and W3, in comparison with vehicle-treated grafts (Fig. 5D). These results indicate that pre-incubation of the vein graft with 3-MA can block the arterial flow-induced excessive autophagy to rescue venous endothelial damage and alleviate subsequent neointimal hyperplasia and that the use of HFU can provide a non-invasive platform to monitor the dynamics of inhibiting restenosis in living animals.

## Discussion

The current study established a non-invasive platform by using HFU and computational simulation to study the roles of mechanical factors in vein graft disease and to explore their relations with pathological cascades. To the best of our knowledge, this is the first paper to provide these findings in animals and enable the prediction of stress overload for excessive autophagy that induces EC damage in vein graft. A suitable range of shear stress benefits angiogenic sprouting^29^, but abnormal shear stress triggers pathological vascular gene expression^30^. The results from both *ex vivo* and *in vivo* experiments suggest that the magnitude of arterial shear stress in the venous segment may still be higher than that is appropriate for the initiation of beneficial remodeling, and hence inducing the excessive autophagy to trigger the cellular apoptosis and pathological remodeling of vein graft disease (Figs 2 and 3). Further validation was performed using the *ex vivo* system, which provides the native microenvironment of vascular tissue without the interference of inflammatory cells in animals *in vivo* to confirm the role of venous-arterial transition of shear stress in EC damage and protein expressions (Fig. 4). Echogenicity carries the information for the density of scanned tissue which can be used to detect tissue remodeling^31^. In accordance to the ultrasonic principles, the increase of echogenicity may arise from alterations in intracellular structures, which could be caused by the accumulation of LC3 puncta and double membrane due to excessive autophagy, or by the changes of cellular shape and density in apoptotic cells.

The modulation of autophagy has been a target for cardiovascular therapy over the past decade^14^. In atherosclerotic plaques, autophagy is protective and contributes to plaque stability by degrading damaged intracellular materials in smooth muscle cells (SMC)^14^, but excessive stimulation of autophagy results in autophagic death of ECs or SMC during atherogenesis^32^. In the ischemic heart, the increase in cardiomyocyte death following pharmacological inhibition of autophagy indicates the pro-survival role of autophagy in mild ischemic stress^16^. However, in cultured neonatal cardiomyocytes, the inhibition of autophagy with 3-MA increased cell viability under ischemic/reperfusion condition. Therefore, autophagy formation has different roles under different conditions, and the methods used to alter autophagy in various target cells are important for the treatment of vascular diseases. In the current study, the excessive autophagy and apoptosis of venous ECs were triggered by the transition of venous-to-arterial shear stress and the consequent inflammatory responses. The co-expression of LC3 and p62 are essential in normal autophagic cycling to degrade the cytoplasmic cargo by hydrolytic enzymes through the lysosomal machinery. The decrease in p62 and increase in LC3 indicated that excessive autophagy in venous ECs might result in cell death by autophagy accumulation. Among various autophagy-related molecules, Beclin 1 is a critical component in the class III PI3 kinase complex that induces the formation of autophagosomes. The abundance of Beclin 1 can be an important determinant of autophagic activity, and can either ensure survival or trigger cell death^12^. The autophagic processes are triggered when Beclin 1 dissociates from B-cell lymphoma (Bcl)-2 and Bcl-X(L)^27^,^28^. Then, the binding of Beclin 1 to Bcl-2 and Bcl-X(L) family members inhibits autophagy to cause pro-survival signals. Beclin 1 also inhibits vesicle processing in the late autophagic cascade to lead to cell death^15^. Our *in vivo* results showed supportive evidence for the dissociation of Beclin 1 complex in venous EC under arterial environment and the rescue potential by transient autophagy inhibition (Supplementary Fig. S6).

Our recent publication^21^ suggests that the increases in echogenicity in injured bone are positively correlated to tissue inflammatory responses, particularly COX-2- or 5-LO-mediated signal pathways. In the present study, immunohistological staining revealed significant increases in protein expression of COX-2, but not BLT1 (receptor of leukotriene B4 for 5-LO signaling), in the venous endothelium at 3 weeks after surgery (Supplementary Fig. S8). Although it has been reported that arteriovenous shunt in mice up-regulates the expression of inflammatory genes (such as E-selectin and VCAM1) and increases the presence of neutrophils and monocytes/macrophages in the vascular wall^33^, we did not observe increases of ICAM1 and VCAM1 in the venous and/or arterial endothelium after ALSS perfusion for 24 hrs (Supplementary Fig. S9). ALSS-induced inflammatory responses was further confirmed by the augmented expression of other cytokines, such as IL-1β and TNF-α (Supplementary Fig. S10). We did not perform experiments to rescue the vein graft failure by direct inhibition of COX-2 because of the apoptotic effects of the non-selective (e.g., indomethacin) or selective COX-2 inhibitors (e.g., Celecoxib)^34^. We demonstrated that short-term pretreatment with an autophagy inhibitor (3-MA) attenuated arterial flow-induced excessive autophagy in venous ECs (Figs 4 and 5) and alleviated the subsequent neointima formation (Fig. 5). However, 3-MA pretreatment only attenuated the COX-2 expression *ex vivo* (Fig. 4C), but had little effect for COX-2 (Fig. 5C) and TNF-α (Supplementary Fig. S11) *in vivo*. The discrepancy may due to the presence of the multifaceted inflammatory components in the blood stream. The administration of 3-MA must be performed carefully because the results of autophagy modulation in caridac diseases have been controversial. It has been reported that induction of autophagy by rapamycin treatment is cardioprotective, but the administration of rapamycin immediately before acute myocardial ischemic/reperfusion reduced cardiac function and increased cell necrosis^15^. It is documented that rapamycin regulates multiple physiological processes and has limited success to complete blockade of the mTOR pathway^35^,^36^,^37^. mTOR is well known to regulate cell autophagy, proliferation, inflammation and death. However, dual effect on modulating mTOR expression or activation always results in different outcomes in various cardiovascular cells. Other specific autophagy inducers via inhibiting of mTOR-relative signals in mTORC1 and mTORC2 (such as PP242 and Torin1) should be used for studying the role of mTOR in excessive autophagy. In addition, altering the physiological autophagy regulation in the whole body by phamacological inhibitors should be careful. The autophagy inhibitor 3-MA reduced the cardiac fibrosis after cardiac pressure overload^38^, but long-term inhibition of autophagy has side effects on macrophages and SMCs^39^. In the current study, the transient treatment of the vein graft with an autophagy inhibitor prior to the *in vivo* venous-to-arterial shear stress transition blocked the autophagic cell death and inflammatory cascades in graft segment. Thus, the pretreatment of grafting vien for excessive autophagy inhibition may provide a good therapuetic strategy to modulate local autophagy activity. Other anti-inflammatory drugs (e.g., asprin and dexamethasone) or anti-cancer drugs (e.g., dichloroacetate) are also used to decrease neointima formation and proinflammatory responses in vein graft surgery^40^,^41^. The efficacy of these treatments or different combinatorial approaches remains to be determined in the future.

**Figure 6:**
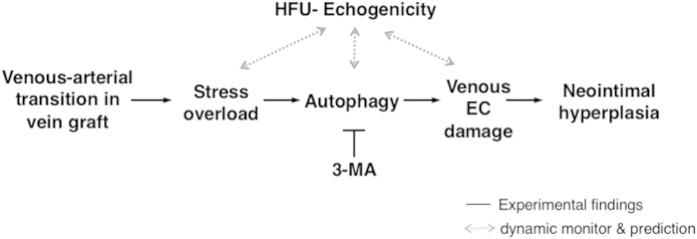
Summary diagram on the monitoring of vein graft progressions by HFU-echogenecity. The prediction of mechanical overload with the consequent induction of EC damage is confirmed by the triggering of autophagic cell death, which can be prevented by the transient administration of 3-MA prior to the venous-arterial transition.

In summary, this study has utilized an integrative approach to elucidate the mechanical and molecular bases of the shear stress-induced failure of vein grafts, which is an important clinical problem. The combination of HFU scanning and computational simulation allows the prediction and detection of venous EC damages triggered by excessive autophagy *in vivo* and *ex vivo* (summarized in Fig. 6). The HFU in the current platform provides sub-tissue resolution for measuring the structure changes in vessels, and also allows for the continuous monitoring of blood flow and echogenicity during stenosis. Our computation of stress distribution from the measured material properties of vessel and the prediction of mechanical overload that would cause potential endothelial damage. Our results indicate that alterations of shear stress play a key role in venous EC remodeling. The finding of inflammation and excessive autophagy in causing cell death provides mechanistic insights into the mechano-responses and the pathological role of excessive autophagy in vein graft stenosis. The alleviation of vein graft stenosis by 3-MA pretreatment indicates its potential for the development of therapeutic approaches to locally modulate autophagy overactivation. Taken together, the usage of HFU echogenicity in conjunction with dynamic stress computation to evaluate vascular disease progression and treatment efficacy may contribute to the development of novel diagnostic and prognostic tools in clinical applications.

## Methods

### Bypass-grafting animals

8-week-old Sprague-Dawley (SD) rats provided by the Animal Center at National Cheng Kung University, with approval from the Institutional Animal Care and Use Committee (IACUC), were used to establish bypass-grafting animal model, including sham (N = 10), carotid artery-to-vein (CAV, N = 12), and carotid artery-to-artery (CAA, N = 12) operations. The methods used on animals were conducted in accordance with IACUC approval guidelines. Before surgery, systemic anesthesia was performed on rats with 0.1 mL/100 g Zoletil 50 (Virbac, France) by intraperitoneal injection. End-to-end anastomosis was performed to create the vein graft model in rats (Supplementary Fig. S1).

### HFU measurements and prediction of stress overload

To monitor the dynamic changes in vein graft stenosis at early stages in living animals, a HFU system was used to scan the detailed vascular structures via B-mode images and to collect information on blood flow using an ultrasonic Doppler device (Supplementary Fig. S2A). The HFU system comprised of a 30-MHz transducer was used to generate and receive ultrasound signals as described in a previous study^21^. The series of cross-sectional images were acquired to monitor the dynamic changes in vascular structures from proximal to distal sites of vessels with 50-μm intervals at post-surgery weeks 1, 2, and 3 (W1, W2, W3). The stenosis progression was calculated from the changes of lumen size in HFU images. The vascular structures during vein graft stenosis were further analyzed by echogenicity to indicate the structure properties of the scanned tissue.

The 3D vessel geometry was reconstructed using MATLAB software (MathWorks, Inc., USA) (Supplementary Fig. S2B), and a commercial software for finite-element analysis (ANSYS Inc., USA) was used to apply 50-μm meshes to the vascular structures to predict the mechanical responses. The simulation setting parameters for flow velocity was obtained from the Doppler ultrasound, and the mechanical properties of the arterial (common carotid artery) and venous (external jugular vein) intima layers were measured by atomic force microscopy (AFM) (NanoWizard II, JPK instruments, Germany) to acquire precise boundary conditions for solid finite-element modeling. The AFM indentation on the lumen surface of freshly isolated normal vessels showed that the Young’s modulus of vein (~0.67 kPa) was significantly lower than that of the artery (~3.49 kPa), thereby indicating a lower stiffness for venous than arterial endothelium (Supplementary Fig. S2C). To determine the yield stress for the prediction of endothelial damage area, the von-Mises stress, which indicates the normal (in perpendicular direction) and shear forces acting on the endothelium, was calculated based upon the combination of the appropriate Young’s modulus and hemodynamic simulation.

### *Ex-vivo* arterial flow perfusion and autophagy inhibition by drug pretreatment

The descending aorta (artery) and inferior vena cava (vein) were isolated from Sprague-Dawley rats (450–500 g) and mounted on perfusion catheters to apply ALSS or VLSS for 1, 3, 6, 12, and 24 hrs (Supplementary Fig. S3A). Trypan blue dye in the lumen was used to visualize the artery and vein subjected to the perfusion induced by the application of shear stress (Supplementary Fig. S3B). The HFU system was also used to monitor the echogenicity during the application of shear stress. After the *ex vivo* application of shear stress, the integrity of endothelium was assessed by *en face* staining of specific endothelial markers on the inner surface of the vessel. To inhibit autophagy, the isolated vein was treated with 3-MA (10 μM, Sigma-Aldrich, USA) for 30 min before the application of *ex vivo* flow perfusion. Autophagy in vein graft in rats was also inhibited by treatment prior to the grafting to the artery using the same condition (10 μM of 3-MA for 30 min).

### Histological assessments of vein graft disease and autophagy formation

The vessels were fixed and embedded in paraffin (Sigma-Aldrich, USA) or frozen tissue matrix (OCT, Leica biosystems, Germany) for sections. Histological stainings for H&E, elastin stain (Sigma-Aldrich, USA), and Verhoeff’s Van Gieson stain were performed to assess the changes of vascular structure for neointima formation and failure of vein graft. The TUNEL (Roche, Switzerland), immunohistochemical (IHC), and immunofluorescent (IF) stainings were used to determine cell apoptosis and inflammatory responses using specific antibodies against inflammation markers, such as COX-2 (Abcam, UK). Autophagy formation was measured by puncta formation of LC3 (Santa Cruz, USA) and expression patterns of p62/SQSTM1, Beclin 1, Bcl-2, and Bcl-X(L) (Santa Cruz, USA). The CD31 antibody (R&D system) was used to identify the endothelial cells. The ultrastructure of autophagosome was observed by typical double membrane morphologies using a TEM (JEM-1400, JEOL, Japan).

### Statistical analyses

Statistical analyses were performed using one-way analysis of variance (ANOVA) or t-test using the Origin software (version 8.5, Origin Lab). Statistical significance was considered at p < 0.05. All experiments were repeated independently at least three times. The multiple regression analysis was implemented to model the relationship between echogenicity (the dependent or criterion variable) and different tissue inflammation variables, including the expression of LC3, COX-2, and cell death (the independent or predictor variables). To test the contribution of all candidate variables in echogenicity, the backward elimination procedure was implemented by incorporating all candidates into the model and then testing the deletion of each variable or combinations in according to previous study. The F-test of the overall fit (Prob >F) was chosen with p < 0.05 and the adjusted R^2^ > 0.8 as an observed significance factor to affect echogenicity.

## Additional Information

**How to cite this article**: Chang, Y.-J. *et al.* Role of Excessive Autophagy Induced by Mechanical Overload in Vein Graft Neointima Formation: Prediction and Prevention. *Sci. Rep.* **6**, 22147; doi: 10.1038/srep22147 (2016).

## Supplementary Material

Supplementary Video S1

Supplementary Video S2

Supplementary Video S3

Supplementary Information

## Figures and Tables

**Table 1:** Multiple regression analysis between echogenicity and histological variables.

Contribution variables	Deleted factor	Adj. R^2^	*P* value
LC3 + COX2 + TUNEL		0.973*	7.44E-5
LC3 + TUNEL	COX2	0.977*	4.86E-6
LC3 + COX2	TUNEL	0.924*	1.82E-4
COX2 + TUNEL	LC3	0.803*	3.23E-3
LC3	COX2, TUNEL	0.899*	6.15E-5
COX2	LC3, TUNEL	0.817*	5.14E-4
TUNEL	COX2, LC3	0.528	1.60E-2

In single variable assessment, the echogenicity in HFU images had the highest correlation with the LC3 positive staining (dash underline, n = 8). With considering multiple variables in associating to echogenicity, the double positive staining of LC3 and TUNEL showed highest correlation (solid underline, n = 8).

^*^Significant factor to affect echogenicity, adj. R^2^ > 0.8 and p < 0.05.
