# Corrigendum: Role of Excessive Autophagy Induced by Mechanical Overload in Vein Graft Neointima Formation: Prediction and Prevention

**DOI:** 10.1038/srep31256

**Published:** 2016-11-14

**Authors:** Ya-Ju Chang, Hui-Chun Huang, Yuan-Yu Hsueh, Shao-Wei Wang, Fong-Chin Su, Chih-Han Chang, Ming-Jer Tang, Yi-Shuan Li, Shyh-Hau Wang, Kirk K. Shung, Shu Chien, Chia-Ching Wu

Scientific Reports 6: Article number: 2214710.1038/srep22147; published online: 02 26 2016; updated: 11 14 2016

The original version of this Article contained errors in the affiliations.

‘Institute of Basic Medical Sciences, National Cheng Kung University, Tainan, Taiwan’

was incomplete, and now reads:

‘Institute of Basic Medical Sciences, College of Medicine, National Cheng Kung University, Tainan, Taiwan’

This error has now been corrected in the HTML and PDF versions of this Article.

