# Supplementary figures and images for: Role of Excessive Autophagy Induced by Mechanical Overload in Vein Graft Neointima Formation: Prediction and Prevention
Source: Sci Rep. 2016 Feb 26;6:22147. doi: 10.1038/srep22147 (PMC4768319; doi:10.1038/srep22147)

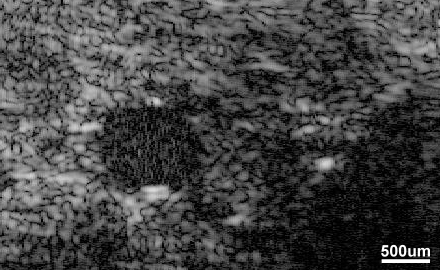

Supplement: Supplementary Video S1 [file srep22147-s1.gif]

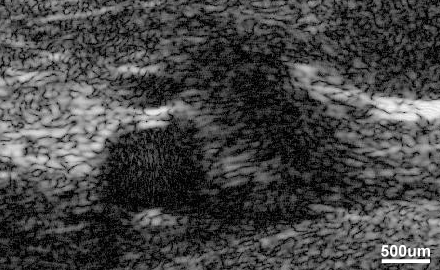

Supplement: Supplementary Video S2 [file srep22147-s2.gif]

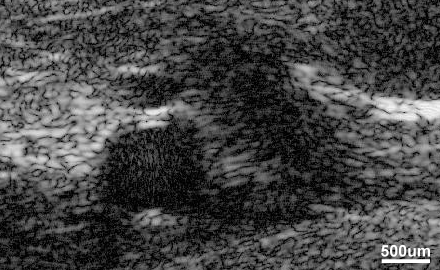

Supplement: Supplementary Video S3 [file srep22147-s3.gif]
