# Supplementary material for: Role of Excessive Autophagy Induced by Mechanical Overload in Vein Graft Neointima Formation: Prediction and Prevention
Source: Sci Rep. 2016 Feb 26;6:22147. doi: 10.1038/srep22147 (PMC4768319; doi:10.1038/srep22147)
Supplement: Supplementary Information [file srep22147-s4.pdf]

Role of Excessive Autophagy Induced by Mechanical Overload in Vein Graft Neointima Formation: Prediction and Prevention

Ya-Ju Chang[1], Hui-Chun Huang[1], Yuan-Yu Hsueh[2], Shao-Wei Wang[3], Fong-Chin Su[3,4], Chih-Han Chang[3,4], Ming-Jer Tang[5], Yi-Shuan Li[6], Shih-Hao Wang[7], Kirk K. Shung[8], Shu Chien[6], Chia-Ching Wu[1, 3, 4, 9*]

[1] Institute of Basic Medical Sciences, National Cheng Kung University, Tainan, Taiwan

[2] Division of Plastic Surgery, National Cheng Kung University Hospital, Tainan, Taiwan

[3] Department of Biomedical Engineering, National Cheng Kung University, Tainan, Taiwan

[4] Medical Device Innovation Center, National Cheng Kung University, Tainan, Taiwan

[5] Department of Physiology, National Cheng Kung University, Tainan, Taiwan

[6] Institute of Engineering in Medicine, University of California, San Diego, CA92093, USA

[7] Department of Computational Science, National Cheng Kung University, Tainan, Taiwan

[8] Department of Biomedical Engineering, University of Southern California, Los Angeles, CA90089, USA

[9] International Research Center for Wound Regeneration and Repair, National Cheng Kung University, Tainan, Taiwan

Information of corresponding author

Address: No. 1, University Rd., Tainan, Taiwan 701.

Tel: +886-6-2353535 ext 5327

Fax: +886-6-2093007

E-mail joshccwu@mail.ncku.edu.tw

* Corresponding author

Supplementary Figure 1

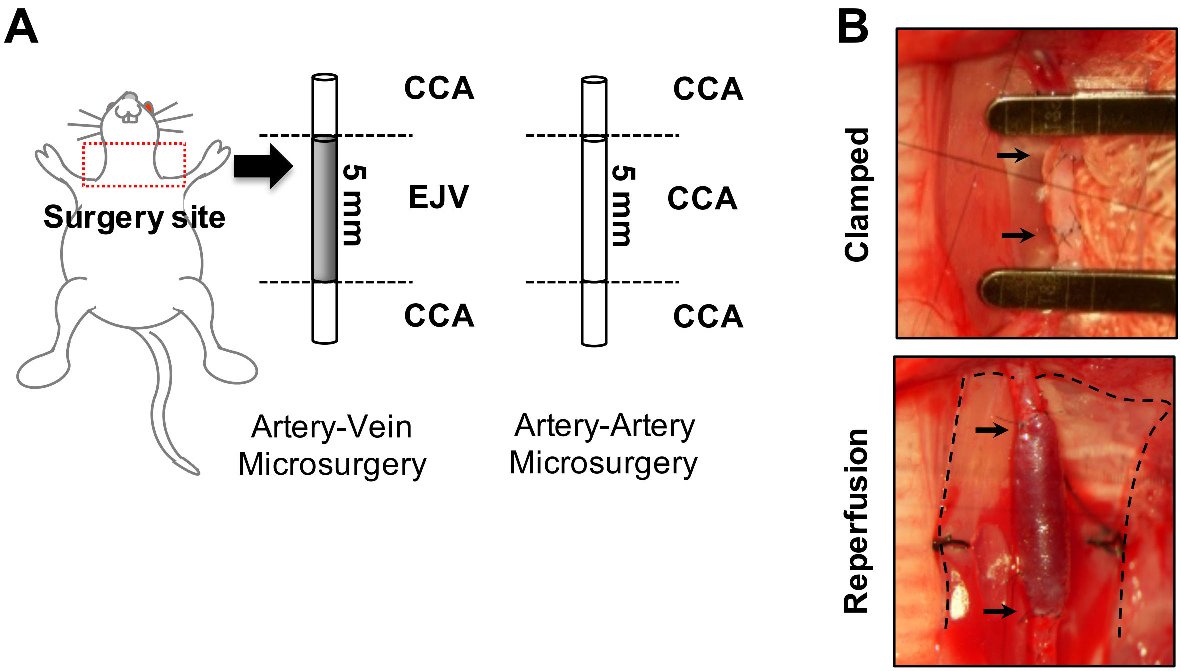

Supplementary Figure S1. vein graft restenosis model was established in Sprague Dawley rats (~800 g). The schematic figure (draw by author of current study) of rat surgery (A). The common carotid artery (CCA) was cut (5-mm length) and replaced with the external jugular vein (EJV). A control group was created by cutting the same artery and suturing it back for the carotid artery-artery group. After suturing and releasing the clamp, the vessel was re-perfused with arterial blood to mimic vein graft surgery in humans (B). The vein graft location was identified by inserting a medical-grade silicone sheet (dash line) under the bypass area.

Supplementary Figure 2

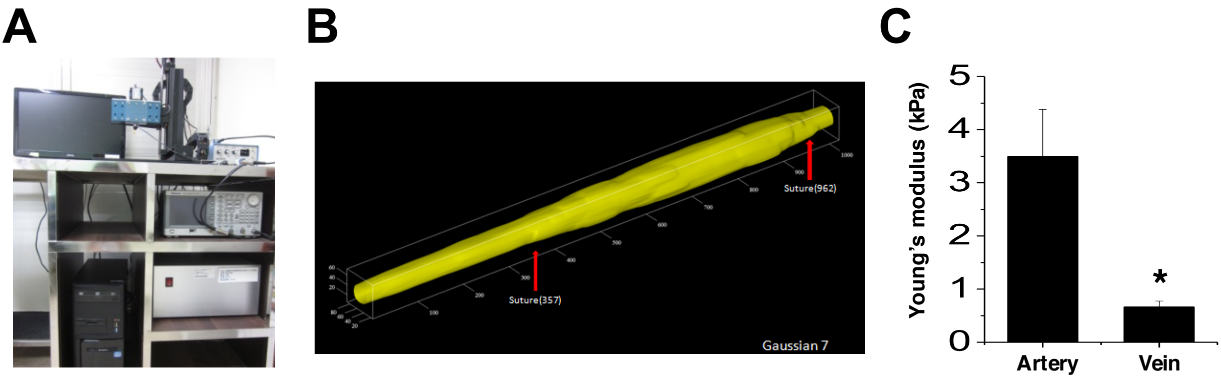

Supplementary Figure S2. Establishment of high-frequency ultrasound (HFU) system and computational platform. System setup of HFU (A). The inner vessel wall was identified from each scanned HFU image and then constructed into 3D vascular structures for simulating the hemodynamics and biomechanical responses (B). The Young's moduli on the inner surfaces of vessels were measured using atomic force microscopy (AFM) and showed significantly softer endothelium in vein than artery (n=4) (C).

Supplementary Figure 3

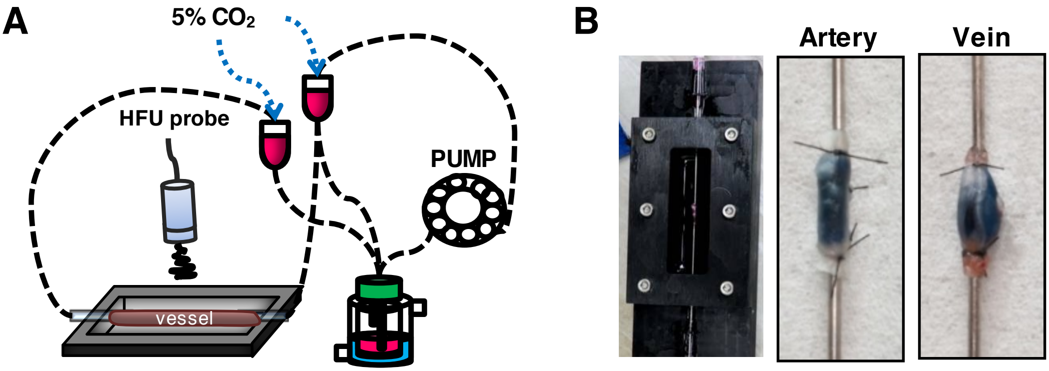

Supplementary Figure S3. *Ex vivo* system was established by harvesting vessels from rats. Schematic diagram of HFU scanning on the *ex vivo* system (A). Differences in luminal size between the artery and vein were observed by injecting the Trypan blue dye into the vascular lumen (B).

Supplementary Figure 4

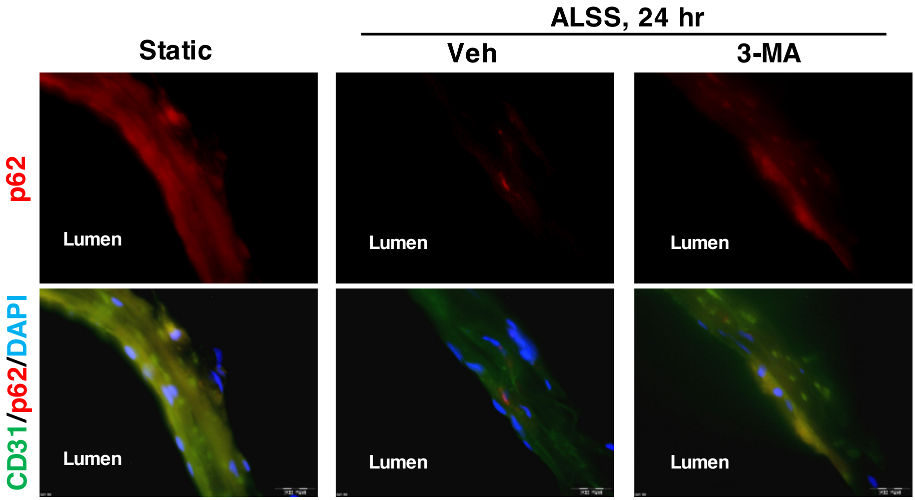

Supplementary Figure S4. Transient pretreatment of 3-MA prevented the decrease of p62/SQSTM1 under ALSS. The excessive autophagy was confirmed by decreasing p62/SQSTM1 in vein after subjected to ALSS for 24hr, but the pretreatment of 3-MA (30min) prior the ALSS application can prevent the p62 degradation.

Supplementary Figure 5

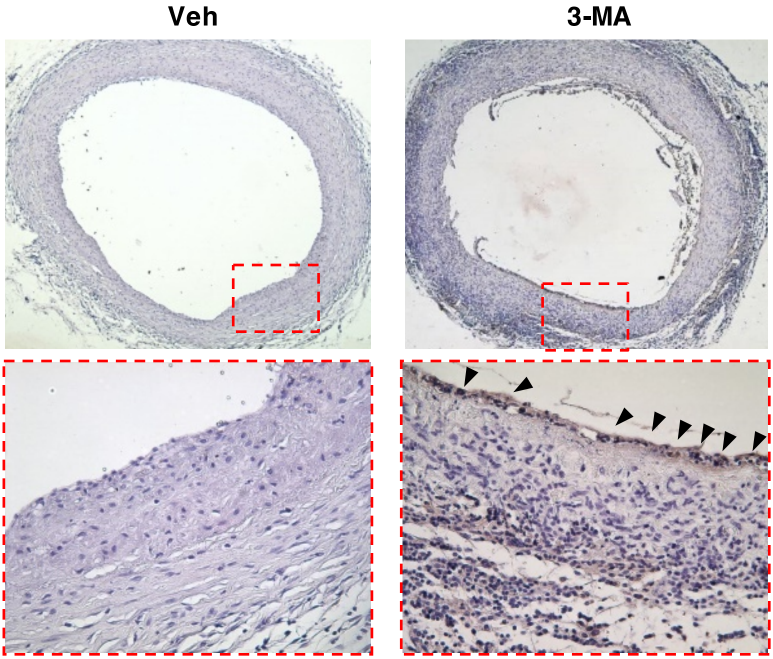

Supplementary Figure S5. The Immunohistochemical (IHC) staining confirmed the preservation of p62/SQSTM1 expression and endothelium in grafted vein by inhibiting the excessive autophagy using 3-MA pretreatment. The squares with dash lines present the enlarged images in the original pictures.

Supplementary Figure 6

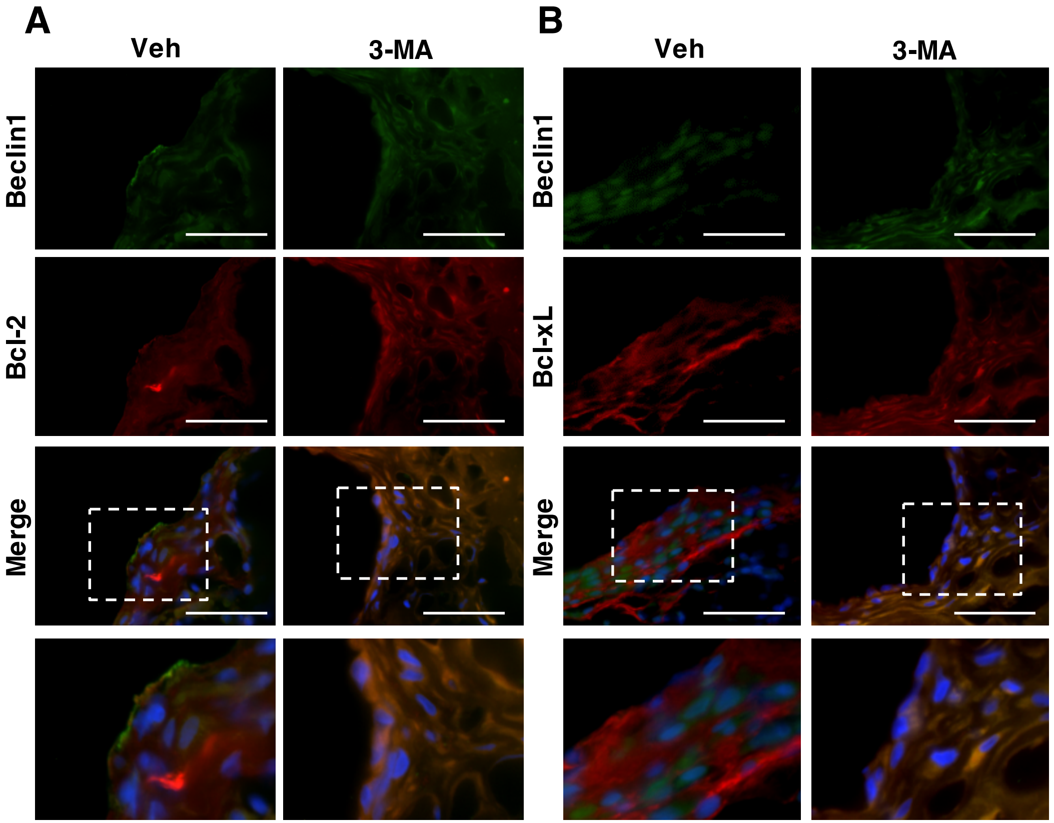

Supplementary Figure S6. Blockage of excessive autophagy prevent the dissociation of Beclin 1 from Bcl-2/Bcl-X(L). Immunofluorescence staining showed the ALSS induced the dissociation of Beclin 1 from Bcl-2 (Veh in A) and Bcl-X(L) (Veh in B) in the vein graft. 3-MA pretreatment prevented the dissociation of Beclin 1 complex. Scale bar: 50 μm.

Supplementary Figure 7

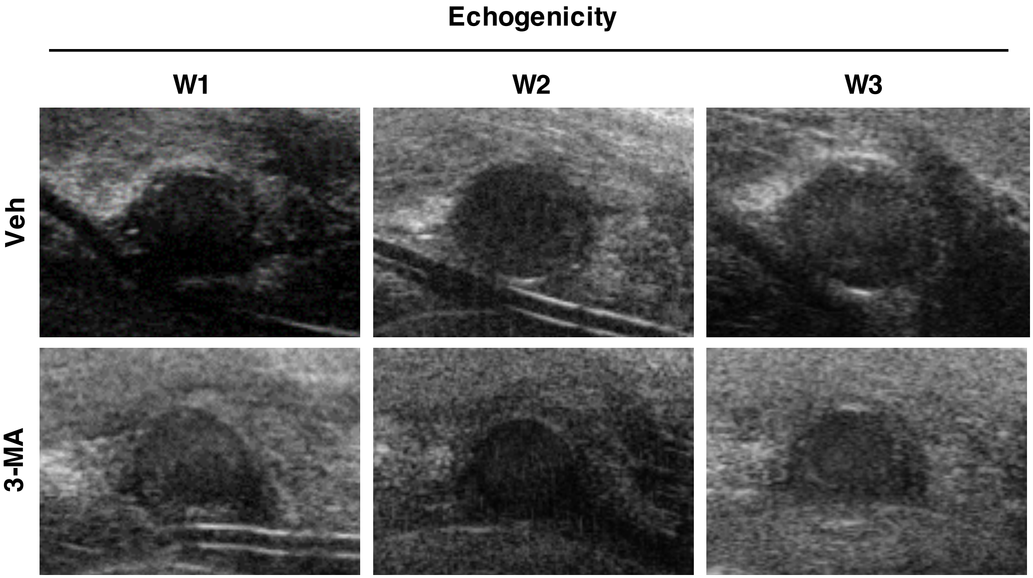

Supplementary Figure S7. Non-invasive observation of inflammatory responses and neointima formation with 3-MA pretreatment using HFU at different time points after surgery. Inhibition of neointima formation and hyper-echogenicity were observed after abolished the excessive autophagy using 3-MA pretreatment.

Supplementary Figure 8

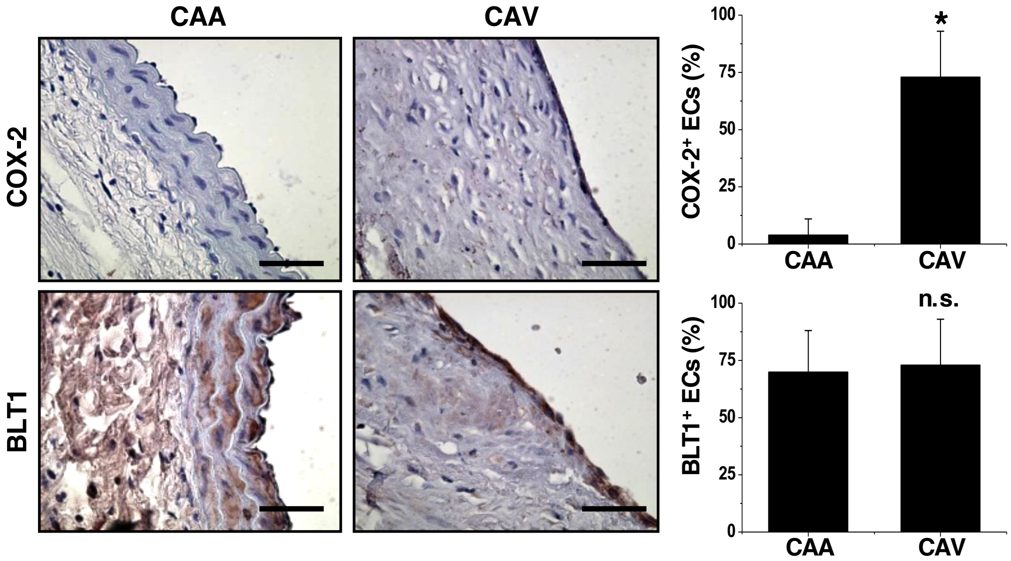

Supplementary Figure S8. Cyclooxygenase-2 (COX-2) signal was triggered in vein grafts by arterial blood flow perfusion. Arterial blood flow triggered the increase of inflammatory response in venous endothelial cells via the COX-2 signaling pathway, but not 5-lipoxygenase (5-LO; BLT1) pathway. Scale bar: 50 μm. *significant difference to the results of CAA, $p<0.05$.

Supplementary Figure 9

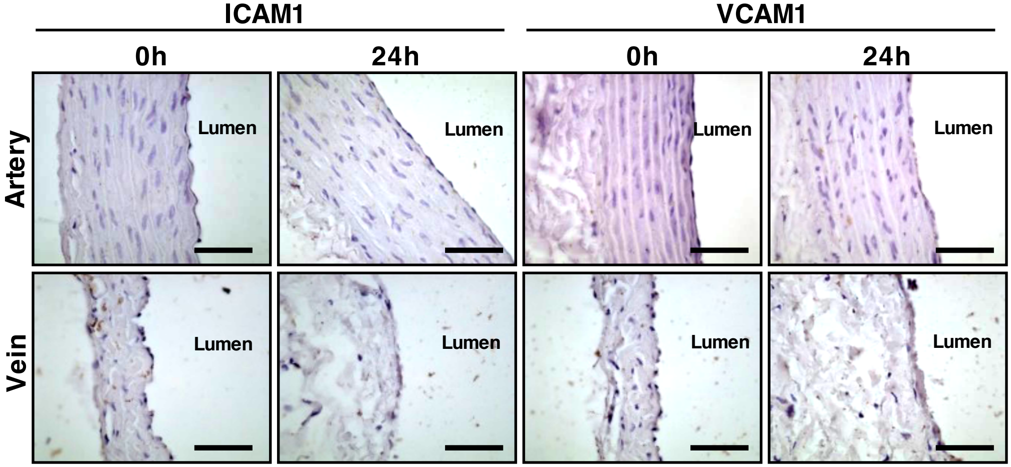

Supplementary Figure S9. Arterial laminar stress (ALSS) did not affect the expression level of intercellular adhesion molecule 1 (ICAM1) and vascular cell adhesion molecule 1 (VCAM1). Both ICAM1 and VCAM1 were not detected in the venous endothelium until the ALSS had been simulated for 24 hrs. Scale bars: 50 μm.

Supplementary Figure 10

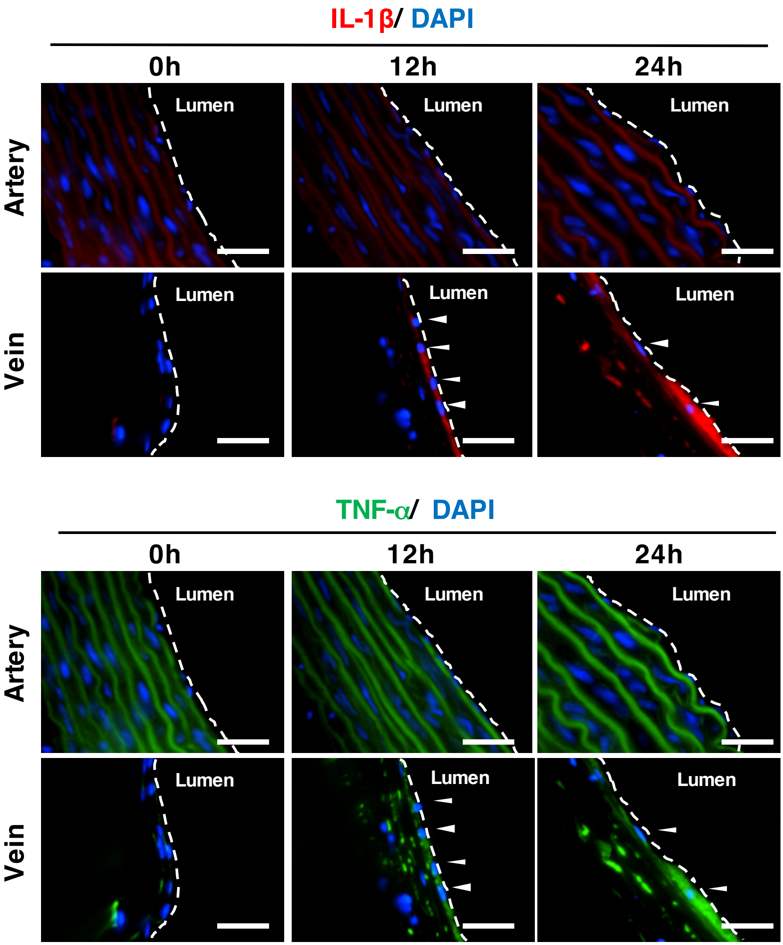

Supplementary Figure S10. ALSS induced the expression of interleukin (IL)-1β and tumor necrosis factor (TNF)-α in veins.
Immunofluorescence staining indicates the ALSS triggered both IL-1β and TNF-α in vein at 12 hrs which were significantly expressed at 24 hrs. Scale bar: 30 μm.

Supplementary Figure 11

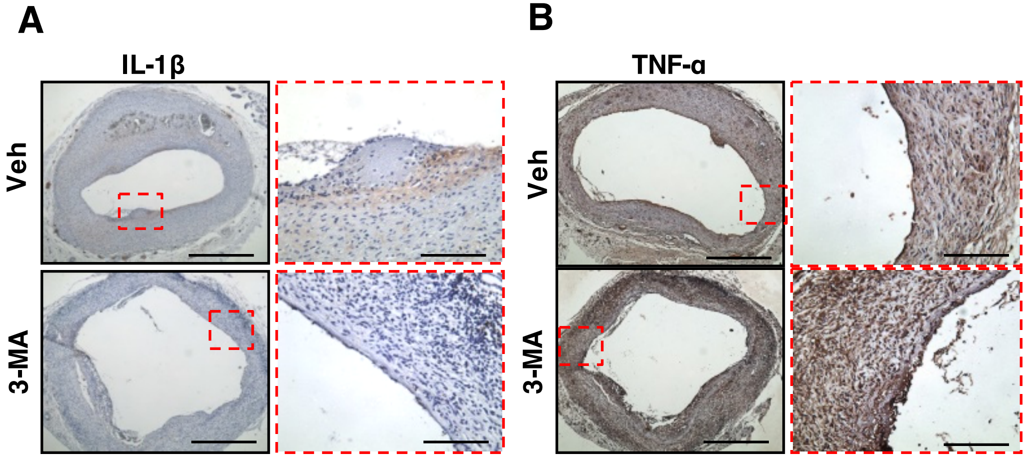

Supplementary Figure S11. 3-MA pretreatment decreased the expression of IL-1β, but not TNF-α, in vein grafts. The IHC staining for IL-1β (A) and TNF-α (B) showed the decrease of IL-1β in 3-MA-treated vein. The squares with dash lines present the enlarged images in the original pictures. Scale bar in original picture: 500 μm; Scale bar in enlarged image: 100 μm.

Supplementary Video Legends

Supplementary Video S1. The serial acquisition of high-frequency ultrasound (HFU) images in the vein graft demonstrated structural changes in different parts of the vessel at 1 week after surgery.

Supplementary Video S2. The pathological changes around the vessel wall was observed by the serial scanning of HFU images in the vein graft at 2 weeks after surgery.

Supplementary Video S3. The pathological changes around the vessel wall was observed by the serial scanning of HFU images in the vein graft at 3 weeks after surgery.
